# Metabolic diversity in a collection of wild and cultivated *Brassica rapa* subspecies

**DOI:** 10.3389/fmolb.2022.953189

**Published:** 2022-11-16

**Authors:** Shuning Zheng, Jędrzej Szymański, Nir Shahaf, Sergey Malitsky, Sagit Meir, Xiaowu Wang, Asaph Aharoni, Ilana Rogachev

**Affiliations:** ^1^ Department of Plant and Environmental Sciences, Weizmann Institute of Science, Rehovot, Israel; ^2^ Department of Molecular Genetics, Leibniz Institute of Plant Genetics and Crop Plant Research (IPK), Seeland, Germany; ^3^ Institute of Vegetables and Flowers, Chinese Academy of Agricultural Sciences, Beijing, China

**Keywords:** *Brassica rapa*, metabolite profiling, mass spectrometry, multivariate data analysis, phylogenetic analysis, specialized metabolism, selective breeding

## Abstract

*Brassica rapa* (*B. rapa*) and its subspecies contain many bioactive metabolites that are important for plant defense and human health. This study aimed at investigating the metabolite composition and variation among a large collection of *B. rapa* genotypes, including subspecies and their accessions. Metabolite profiling of leaves of 102 *B. rapa* genotypes was performed using ultra-performance liquid chromatography coupled with a photodiode array detector and quadrupole time-of-flight mass spectrometry (UPLC-PDA-QTOF-MS/MS). In total, 346 metabolites belonging to different chemical classes were tentatively identified; 36 out of them were assigned with high confidence using authentic standards and 184 were those reported in *B. rapa* leaves for the first time. The accumulation and variation of metabolites among genotypes were characterized and compared to their phylogenetic distance. We found 47 metabolites, mostly representing anthocyanins, flavonols, and hydroxycinnamic acid derivatives that displayed a significant correlation to the phylogenetic relatedness and determined four major phylometabolic branches; 1) Chinese cabbage, 2) yellow sarson and rapid cycling, 3) the mizuna-komatsuna-turnip-caitai; and 4) a mixed cluster. These metabolites denote the selective pressure on the metabolic network during *B. rapa* breeding. We present a unique study that combines metabolite profiling data with phylogenetic analysis in a large collection of *B. rapa* subspecies. We showed how selective breeding utilizes the biochemical potential of wild *B. rapa* leading to highly diverse metabolic phenotypes. Our work provides the basis for further studies on *B. rapa* metabolism and nutritional traits improvement.

## 1 Introduction

*Brassica rapa* (*B. rapa*) is an economically important crop species of the genus *Brassica* and is widely cultivated and consumed worldwide. During the long history of selective breeding, it reached an enormous morphological diversity and a wide range of useful purposes, including leafy vegetables (e.g., Chinese cabbage, pak choi, and mizuna), inflorescence vegetables (e.g., caixin and broccoletto), floral shoot and stem vegetables (e.g., purple caitai and turnip top), enlarged root vegetables or fodders (e.g., turnip), as well as oilseed crops (e.g., yellow sarson). Due to its strong adaptability, short growth period, high yield, unique flavor, and nutritional benefits, *B. rapa* is increasingly popular worldwide (Salehi et al., 2021).

*Brassica* vegetables have been widely acknowledged for their beneficial effects on human health. Epidemiological studies have indicated that increased consumption of *Brassica* vegetables is strongly associated with a reduced risk of cancer, cardiovascular disease, diabetes, and immune dysfunction (Raiola et al., 2018; Salehi et al., 2021). These health-related properties have been attributed to nutrients and health-promoting phytochemicals, such as *Brassica*-specific glucosinolates, carotenoids, vitamins, and phenolic compounds (Paul et al., 2019). Glucosinolates and their breakdown products have been reported to reduce the risk of lung, colon, and other types of cancer (Mandrich and Caputo, 2020). Phenolic compounds in plants possess potential health-promoting effects, including antioxidant, anti-inflammatory, anti-microbial, anti-obesity, and anti-tumour activities (Cao et al., 2021).

The potential activity and bioavailability of dietary phytochemicals in *B. rapa* depend on the chemical structure, modifications, and content. Most previous studies on *B. rapa* have focused on specific classes of targeted compounds, such as glucosinolates, organic acids, or phenolic compounds. For example, glucosinolate profiles in different *B. rapa* varieties have been reported (Liu et al., 2020; Zou et al., 2021). Phenolic compounds have been investigated in turnip (Chihoub et al., 2019), Chinese cabbage (Managa et al., 2020), pak choi (Jeon et al., 2018; Yeo et al., 2021), and mizuna (Kyriacou et al., 2021), establishing flavonoids and hydroxycinnamic acids as main phenolic compounds. However, the morphological, flavor, and taste diversity of *B. rapa*, suggest much wider metabolic complexity, potentially including new interesting compounds and biochemistry.

**FIGURE 1:**
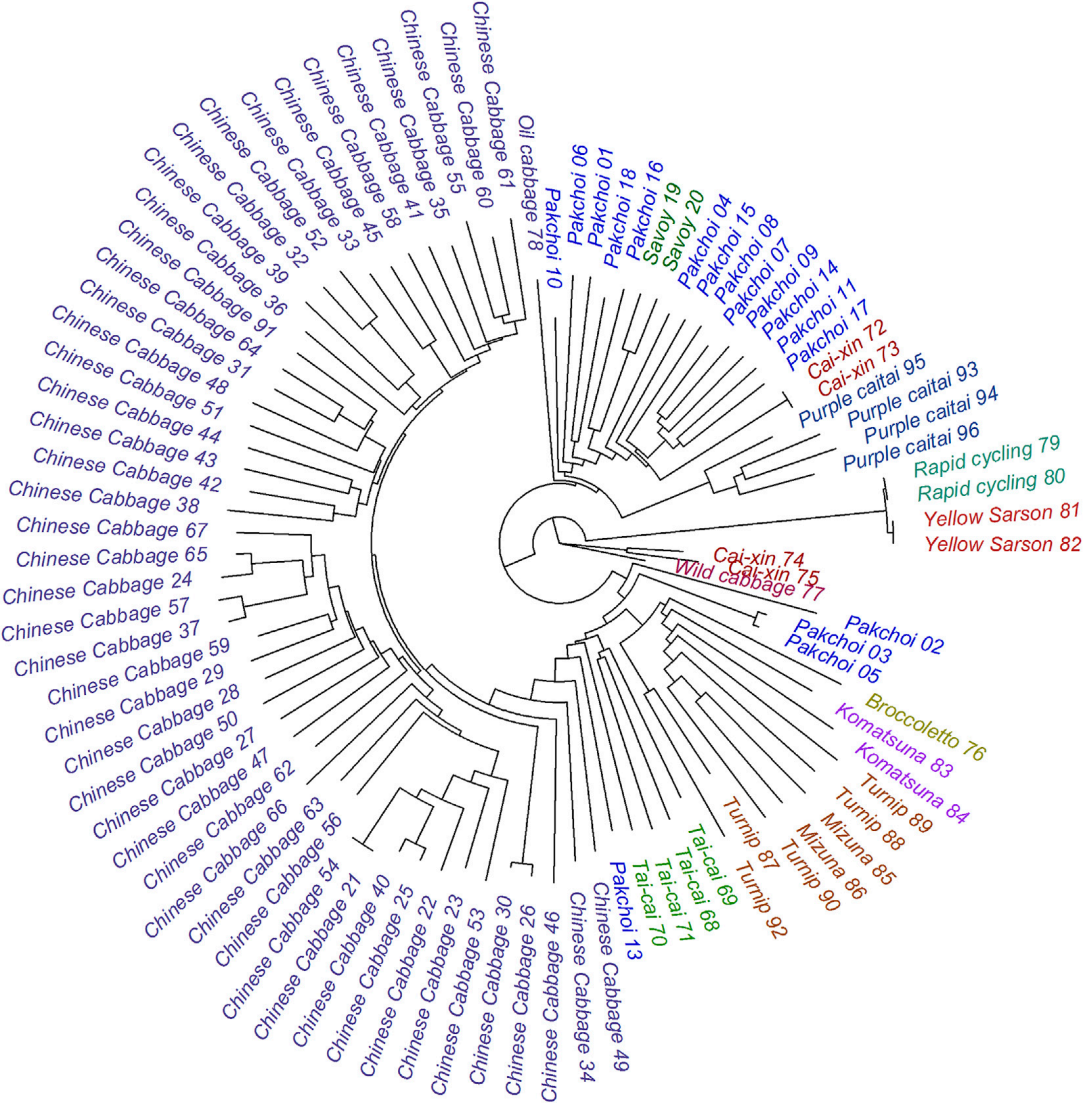
The phylogenetic distance tree of the 102 analyzed *B. rapa* accessions based on the DNA sequence variation—SNPs with MAF >0.05 as described by Cheng et al. (2016). The tree is rooted in the wild-type cabbage genotype. Classification of the sub-species is represented by the color code.

In the present study, we performed comprehensive metabolic characterization of 102 representative *B. rapa* genotypes, covering 14 subspecies and their individual accessions exhibiting a wide variety of morphological traits (Figure 1). Clustering analysis revealed similarities of accessions and metabolites in metabolic composition and chemical structure, respectively. Furthermore, we carried out phylogenetic analysis and assessed the relationship between metabolic composition and genetic relatedness of various *B. rapa* accessions. This highlighted the biochemical effects of selective breeding of *B. rapa*.

## 2 Materials and methods

### 2.1 Chemicals

All solvents were of HPLC grade. Methanol and acetonitrile were purchased from Merck KGaA (Darmstadt, Germany). Formic acid was purchased from J.T. Baker (Germany). Ultrapure water was produced using a Milli-Q water purification system (Millipore, Bedford, MA, United States).

### 2.2 Plant material

We selected 102 representative *B. rapa* genotypes belonging to 14 main *B. rapa* subspecies groups for metabolite profiling analysis, including accessions of Chinese cabbage, pak choi, caixin, turnip, savoy, mizuna, taicai, komatsuna, purple caitai, rapid cycling, yellow sarson, broccoletto, oil cabbage, and wild cabbage (see Table 1 and Figure 1). All of selected *B. rapa* accessions were previously genotyped (Cheng et al., 2016), and SNPs with MAF (minor allele frequency) > 0.05 were retrieved, as described by Cheng et al. (2016). Leaf samples were obtained from the Institute of Vegetable and Flowers, Chinese Academy of Agricultural Sciences (IVF-CAAS, Beijing, China). All plants were cultivated in a greenhouse under the same growth conditions in the fall of 2012. Fifty days after seeding, two or three fresh leaves (about 15–20 g) of uniform size and free from decay and mechanical damage were harvested, snap-frozen in liquid nitrogen, and lyophilized. The freeze-dried samples were then ground into fine powder and stored at −80°C until further analysis. Three biological replicates were taken for each genotype.

**TABLE 1:** Summary of the 102 *B. rapa* accessions in this study.

Accession name	Subspecies	Sample number	Samples in total
Chinese cabbage	ssp. *Pekinensis*	#21–#67, #91	48
Pak choi	ssp. *Chinensis*	#01–#18	18
Purple caitai	ssp. *Chinensis* var. *Purpurea*	#93–#102	10
Turnip	ssp. *Rapa*	#87–#90, #92	5
Caixin	ssp. *Parachinensis*	#72–#75	4
Taicai	ssp. *Chinensis* var. *Tai-tsai*	#68–#71	4
Savoy	ssp. *Narinosa*	#19–#20	2
Rapid cycling		#79–#80	2
Yellow sarson	ssp. *Tricolaris*	#81–#82	2
Komatsuna	var. *Pervidis*	#83–#84	2
Mizuna	ssp. *Nipposinica*	#85–#86	2
Broccoletto	ssp. *Broccoletto*	#76	1
Wild cabbage		#77	1
Oil cabbage		#78	1

### 2.3 Metabolite extraction and sample preparation

Powdered *B. rapa* leaf material (200 mg) was extracted with 80% (v/v) aqueous methanol containing 0.1% (v/v) formic acid by 20 min sonication at room temperature. The extract was centrifuged at 13,000 g for 15 min, and the supernatant was filtered through a 0.22-μm syringe PVDF filter and transferred to an HPLC vial for LC-MS analysis.

### 2.4 UPLC-PAD-QTOF-MS/MS analyses

Non-targeted metabolite analysis was performed on a UPLC-qTOF system (Waters Synapt) with the UPLC column connected in-line to a PDA detector and then to the MS detector (Synapt, Water Corp, Manchester, United Kingdom) equipped with electrospray ionization (ESI) source. Chromatographic separation was carried out using an UPLC BEH C18 column (100 × 2.1 mm i. d, 1.7 µm, Waters Acquity). The mobile phase consisted of two solvents: 0.1% formic acid in acetonitrile/water (5:95, v/v) (A) and 0.1% formic acid in acetonitrile (B). The linear gradient program was as follows: 100%–72% A over 22 min, 72%–60% A over 0.5 min, 60%–0% A over 0.5 min, held at 100% B for a further 1.5 min, then returned to the initial conditions (100% A) in 0.5 min and conditioning at 100% A for 1 min. The flow rate was 0.3 ml min^−1^, and the column temperature was maintained at 35 °C. The injection volume was 4 µl. UV-vis spectra were recorded in the range of 210–500 nm.

The MS conditions were as follows: capillary voltage of 3.0 kV, cone voltage of 28 V, source temperature of 125°C, desolvation temperature of 275°C, desolvation gas flow rate of 650 L h^−1^, and cone gas flow rate of 25 L h^−1^. Nitrogen was used as desolvation and cone gas, and argon was utilized as the collision gas. Data were acquired in MS^E^ mode from *m/z* 50 to 1,500 in centroid mode at negative ion mode, comprising two interleaved full scan acquisition functions: the low energy function and the high energy function. The low energy function employed collision energy at 4 eV to acquire accurate mass data for intact precursor ions. For the high energy function, a collision energy ramp of 10–35 eV was applied for fragmentation information. The MS system was calibrated using sodium formate. Leucine enkephalin was used as a reference lock-mass compound to ensure mass accuracy. The [M-H]^-^ ion at *m/z* 554.2615 was detected via the independent LockSpray™ channel. A mixture of 15 standard compounds, injected after each batch of 10 biological samples, was used for instrument quality control. MassLynx software version 4.1 (Waters) was used to control the instrument and calculate accurate masses.

### 2.5 Data processing and statistical analysis

LC-MS raw data files were converted to NetCDF format using MassLynx DataBridge (version 4.1; Waters Corp.). Peak picking, retention time correction, and alignment were then performed using R packages XCMS (Smith et al., 2006) and CAMERA (Kuhl et al., 2012). Data normalization, analysis, and visualization were performed using R 3.1.2 (Ihaka and Gentleman, 1996). The relative peak intensities were normalized to the median intensity of each chromatogram and subsequently scaled between the minimum non-zero and the maximum value of the original dataset. Hierarchical clustering analysis (HCA) in heat map was performed using Euclidean distance and average linkage on Z-transformed variables (either rows/metabolites or columns/samples). Significant differences in the accumulation of metabolites in measured accessions were identified by one-way ANOVA (Ritchie et al., 2015) with FDR ≤0.05 and followed by Tukey’s HSD test (Supplementary Table S2).

### 2.6 Phylogenetic distance analysis

The neighbor-joining tree was constructed by the BioNJ algorithm (Gascuel, 1997) using J-C distance in PHYLIP 3.6 software (Felsenstein, 2004) and all SNPs with MAF >0.05. The tree was rooted using a midpoint method (Farris, 1972). Phylogenetic signal was computed in a “picante” R package (v1.8.2 2020; Kembel et al., 2010) using Blomberg’s K statistics (Blomberg et al., 2003) on the background of a Brownian motion model of the trait evolution. The significance of the phylogenetic signal was obtained in 9,999 random permutations of the phylogenetic tree labels (Supplementary Table S2).

### 2.7 Clustering of metabolites

For all metabolites SMILES codes have been obtained and translated to the standard molecular fingerprints as described by Faulon et al. (2003) using the rcdk R package (v3.6.0 2021; Guha, 2007). The Tanimoto similarity has been calculated according to the method of Fligner et al. (2002) and the result has been displayed as a hierarchical clustering tree using the complete agglomerative linkage method. Metabolites sharing the same fingerprint have been grouped and treated as one single compound.

## 3 Result and discussion

### 3.1 Strategy for metabolite identification

To comprehensively characterize the metabolome of *B. rapa* leaves, a total of 102 representative *B. rapa* genotypes, belonging to 14 major *B. rapa* subspecies groups, were selected to cover their large genetic and phenotypic variations (Figure 1). Untargeted metabolite analysis was performed using UPLC-PDA-QTOF-MS/MS and representative total ion chromatograms (TIC) of five *B. rapa* accessions are shown in Supplementary Figure S1. Out of 7,286 quantified mass features, a total of 346 metabolites were identified at different confidence levels. In our study, two metabolite identification strategies were used: 1) high-confidence metabolite identification based on authentic standards and 2) putative identification based on literature and public databases. In total, 37 metabolites were identified with the “high confidence” strategy through comparison of retention time (RT), UV/Vis spectra, accurate mass, isotopic distribution, and fragmentation pattern with those of authentic standards using the WEIZMASS library, a reference spectral library comprising spectra of 3,540 highly pure plant metabolites (Shahaf et al., 2016). Of these, 13 metabolites were identified for the first time in *B. rapa*. Respectively, 309 metabolites were putatively identified in *B. rapa* leaves by surveying the literature and public databases (KNapSack, DNP, Massbank, KEGG, and ReSpect). Metabolites previously reported in the Brassicaceae family were collected in a custom reference database that included metabolite names, molecular formulas, molecular weight, chemical structures, biological sources, and literature or database resources. Mass features following XCMS and Camera clustering were first searched against this reference database using a homemade script. The accurate mass of the molecule and adduct ions as well as their isotope distribution patterns were considered as main search parameters. Next, the structural information from UV/vis spectra and mass fragmentation patterns of the hits were used for putative identification. In this study, a large number of metabolite isomers were identified and discriminated in *B. rapa* leaves based on retention time and/or MS fragments (see Supplementary Material).

### 3.2 Chemical complexity of the metabolic profiles

**FIGURE 2:**
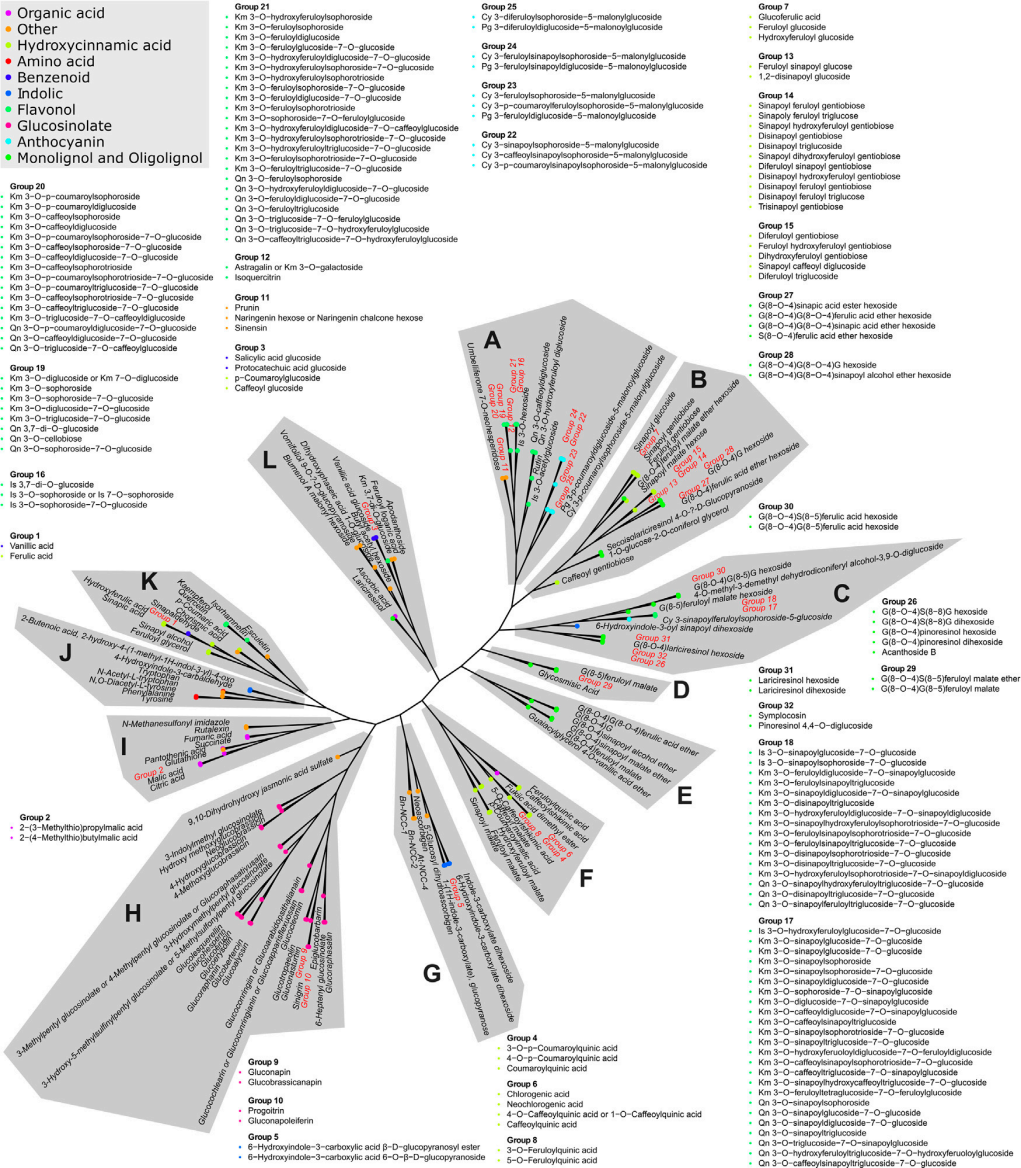
Tanimoto similarity-based clustering tree representing the chemical complexity of the obtained metabolic profiles. The distance matrix is clustered using the complete linkage method. The classification of chemical compounds, according to the Dictionary of Natural Products (https://dnp.chemnetbase.com), is represented by the color code. Twelve major compound cluster groups are marked by letters A to L. Metabolites exhibiting identical fingerprints are represented by numbered groups from 1 to 32. Members of all groups are listed in the proximity of their original position.

The 346 putatively identified metabolites belong to various chemical classes, including 105 flavonols, 93 hydroxycinnamic acid derivatives, 51 monolignol and oligolignol derivatives, 33 glucosinolates, 14 anthocyanins, 10 organic acid, 8 indolics, 5 benzenoids, 3 amino acids, and 24 others. These metabolites are mostly products and intermediates of specialized metabolism pathways associated with nutritional and health-promoting effects of *B. rapa* as well as flavor and aroma (Salehi et al., 2021). To our knowledge, 184 of the detected metabolites were identified in *B. rapa* for the first time. The complete list of all identified metabolites with respective chemical, analytical, and biological descriptors is provided in Supplementary Table S1. Tanimoto similarity analysis showed that the identified metabolites are linked to twelve major clusters based on their chemical structure (Figure 2). Expectedly, most of these clusters were enriched by a specific class of metabolites. However, the non-biased grouping highlighted also four clusters containing metabolites of diverse classes (clusters I, J, K, and L) and included mostly precursors and intermediates upstream in the major pathways of specialized metabolism.

### 3.3 Characterization of major specialized metabolite classes in *B. rapa*

All putatively identified metabolites were categorized into ten major chemical classes based on structures and fragmentation patterns. Both the composition and relative content of metabolites varied significantly among measured accessions (Supplementary Table S2), indicating the impact of genetic diversity on the metabolic variation within the *B. rapa* species.

#### 3.3.1 Glucosinolates

Glucosinolates are a group of nitrogen- and sulfur-containing specialized metabolites and are classified into aliphatic, aromatic, and indole glucosinolates, according to whether they originate from aliphatic amino acids, aromatic amino acids or tryptophan, respectively. Glucosinolates contain a *β*-D- glucopyranosyl common core moiety and a variable side chain. Several studies presented the typical MS fragmentation of glucosinolates (Fabre et al., 2007; Francisco et al., 2009). First, based on the common core structure, glucosinolate could produce characteristic fragments at *m/z* 96.96, 195.03, 241.00, 259.01, and 274.99 via the cleavage of bonds on either side of the sulfur atoms. However, not all fragments could always be observed in MS^E^ fragmentation. We used the most abundant fragment ions at *m/z* 96.96 (sulfate anion) and *m/z* 259.01 (sulfated glucose anion) as diagnostic ions to preliminarily check the presence of glucosinolates. In addition, glucosinolates undergo consistent and characteristic neutral losses of sulfur trioxide (SO_3_, 79.96 amu), anhydroglucose (Glc, 162.05 amu), dehydroxythioglucose (SGlc-OH, 178.03 amu), thioglucose (SGlc, 196.04 amu) as well as combined loss of sulfur trioxide and anhydroglucose (Glc + SO_3_, 242.01 amu), parameters that could be used for identification of variable side chains. Finally, the variable side chain could also produce unique fragments. For example, glucohesperin is an aliphatic glucosinolate with a deprotonated molecular ion at *m/z* 464.07 and formula as C14H27NO10S3. The MS fragments showed characteristic fragment ions at *m/z* 79.95, 274.99, 259.01, 241.00, and 195.03. Neutral loss fragments from deprotonated molecular were observed at *m/z* 449.04 (loss of a methyl moiety from the side chain, −15.03 amu), *m/z* 384.11 (loss SO3, −79.96 amu), *m/z* 226.06 (loss Glc + SO3, −242.01 amu) and *m/z* 400.07 (loss of a methylsulfinyl moiety from the side chain, −64.00 amu). In addition, the dimethylsulfinyl fragment ion (*m/z* 400.07) underwent further neutral loss to give the product ions at *m/z* 238.02 (loss of Glc, −162.05 amu), *m/z* 204.03 (loss of SGlc, −196.04 amu) and *m/z* 158.06 (loss of Glc + SO3, −242.01 amu). Therefore, this long-chain methylsulfinylalkyl glucosinolate was tentatively identified as glucohesperin (Supplementary Table S1). Glucohesperin was reported in *Arabidopsis thaliana* (van de Mortel et al., 2012), while it was detected here in *B. rapa* for the first time. In total, 33 glucosinolates were putatively identified, consisting of 24 aliphatic glucosinolates, 4 aromatic glucosinolates, and 5 indole glucosinolates, including most of the glucosinolates reported earlier in *B. rapa* (Liu et al., 2020; Zou et al., 2021). To our knowledge, 16 of the detected glucosinolates were found in *B. rapa* leaves for the first time.

Glucosinolates are well-known for their roles in plant defenses against herbivores and pathogens (Chhajed et al., 2020). In addition, previous studies demonstrated that aliphatic glucosinolates were predominant in *B. rapa*, with gluconapin and glucobrassicanapin being the most abundant (Klopsch et al., 2018). In the present study, gluconapin and glucobrassicanapin were present in all investigated *B. rapa* genotypes. Moreover, yellow sarson accession #82, (here and further in the text “#” denotes genotype number in Supplementary Table S2) and turnip #89 were found to contain the highest contents of gluconapin and glucobrassicanapin, respectively. Meanwhile, the lowest contents of them were found in Chinese cabbage #57 and pak choi #14, respectively. Among all genotypes, the relative contents of gluconapin and glucobrassicanapin had 19,085- and 1,463-fold differences between the highest and the lowest values, respectively, indicating a large variation in glucosinolates. This is in line with previous studies that demonstrated extensive variation in glucosinolates in 113 turnip varieties (Padilla et al., 2007), 91 different *B. rapa* genotypes (Klopsch et al., 2018) and 82 *B. rapa* varieties (Yang and Quiros, 2010). We found various glucosinolates accumulation patterns among genotypes. For example, epiglucobarbarin and glucohesperin were presented in most genotypes, while glucocleomin and glucolesquerellin were highly accumulated only in accession savoy #20, and at lower levels in all mizuna, turnip, and purple caitai accessions. Upon cell disruption, glucosinolates are hydrolyzed to various breakdown products, which possess a wide range of health-promoting properties. Sulforaphane, the active hydrolysis product of glucoraphanin, has attracted attention due to its significant anticancer properties (Haq et al., 2021). We found that its precursor glucoraphanin was highly enriched in yellow sarson #81 and #82 and rapid cycling #79. Also, indole-3-carbinol, derived from the breakdown of glucobrassicin, showed diverse biological properties with anti-atherogenic, antioxidant, anti-carcinogenic, and anti-inflammatory activities (Kim and Park, 2018). The precursor glucobrassicin was found at the highest level in Chinese cabbage #60, which is in line with earlier reports (Padilla et al., 2007; Yang and Quiros, 2010). In the case of aromatic glucosinolates, gluconasturtiin and glucotropaeolin have been reported to be hydrolyzed by the plant enzyme myrosinase to yield phenethyl isothiocyanate and benzyl isothiocyanate, which have anti-cancer and antimicrobial activities (Cao et al., 2021). In this study, we found that accessions savoy #20, komatsuna #83, and pak choi #5 exhibited relatively higher levels of gluconasturtiin as compared with other genotypes (highest in komatsuna). In addition, glucotropaeolin was mainly accumulated in Chinese cabbage #33. Therefore, these *B. rapa* genotypes with high levels of glucosinolates might be used in future health-related applications.

#### 3.3.2 Flavonols

Flavonols are the predominant phenolic compounds in *B. rapa*. Identification of flavonol glycosides was based on their fragmentation pattern (Ferreres et al., 2004; Harbaum et al., 2007; Lin et al., 2011). The breakdown of the O-glyosidic bond is a typical fragmentation of flavonol glycosides. Previous studies indicated that the O-glyosidic bond at the 7-position was the weakest glycosidic linkage in the flavonols molecule (Ferreres et al., 2004). Thus, the first loss usually was the glycose or acyl-glycose moiety at the 7-position, and then the loss of glycose or acyl moieties at position 3. For acylated flavonol glycosides, neutral loss information was used to characterize acyl groups by the losses of 42.01, 146.04, 162.03, 176.05, 178.03, 192.04, and 206.06 amu for acetyl, p-coumaroyl, caffeoyl, feruloyl, hydroxycaffeoyl, hydroxyferuloyl, and sinapoyl, respectively. In addition, losses of 180.06, 162.05, and 120.04 amu from interglycosidic fragmentations suggested sophoroside or sophorotrioside with 1→2 glycosidic linkage (Ferreres et al., 2004; Lin et al., 2011). As an example, compounds 205–208 were found with the same deprotonated molecular ion at *m/z* 977.26 and aglycone ions (*m/z* 285.04 and *m/z* 284.03), suggesting that they were the isomeric kaempferol glycosides. First, for compounds 205 and 206, the fragment ion at *m/z* 815.20 as a base peak was observed due to the loss of a glucosyl moiety (−162.05 amu) at the 7-O position. Another fragment ion at *m/z* 609.15 was due to the further loss of sinapoyl moiety at the 3-position (−206.06 amu). After the loss of a diglucosyl moiety at the 3-position (−324.11 amu), kaempferol aglycone ions were detected. Moreover, compound 205 showed the fragments at *m/z* 489.11 (−120.04 amu) and *m/z* 429.08 (− 180.06 amu) to confirm the sophorosyl moiety. Thus, compounds 205 and 206 were putatively identified as kaempferol 3-O-sinapoylsophoroside-7-O-glucoside and kaempferol 3-O-sinapoyldiglucoside-7-O-glucoside, respectively. For the other two isomers (compound 207 and 208), a fragment ion at *m/z* 609.15 was detected as a base peak due to the simultaneous loss of a glucosyl moiety (−162.05 amu) and a sinapoyl moiety (−206.06 amu) at the 7-position. Further loss of diglucosyl moiety at the 3-position gave rise to the kaempferol aglycone ions. Together with the characteristic fragments of sophoroside, compounds 207 and 208 were putatively identified as kaempferol 3-O-sophoroside-7-O-sinapoylglucoside and kaempferol 3-O-diglucoside-7-O- sinapoylglucoside, respectively (Supplementary Table S1). In this study, a total of 105 flavonols, including 66 kaempferol derivatives, 28 quercetin derivatives, and 11 isorhamnetin derivatives were identified. To the best of our knowledge, 50 *B. rapa* flavonols are reported here for the first time. Among flavonols, three aglycons (kaempferol, quercetin, and isorhamnetin), 16 non-acylated glycosides, 61 monoacylated, and 25 diacylated glycosides were detected. *B. rapa* leaves showed complex flavonols conjugate with different glycosylation and acylation patterns. Some flavonols possess molecular weight above 1,000 Da, and this increased the complexity of metabolite identification. In one example, detailed identification of compound 263, a diacylated quercetin tetra-glycoside with *m/z* 1,317.34, is presented in the Supplementary Material. In the case of non-acylated flavonol glycosides, mono-, di-, and tri-glycosides of isorhamnetin and quercetin as well as mono- to tetra-glycosides of kaempferol were found. Moreover, 86 flavonol glycosides were acylated with acetic, *p-*coumaric, caffeic, sinapic, ferulic, hydroxyferulic, and hydroxycaffeic acids. We found that mono-acylated glycosides widely existed as kaempferol, quercetin, and isorhamnetin glycosides. However, diacylated glycosides were only present as quercetin tetraglycosides as well as tri-, tetra-, and pentaglycosides of kaempferol. In good agreement with previous reports (Chihoub et al., 2019; Wiesner-Reinhold et al., 2021), kaempferol glycosides were the most diverse flavonols derivatives in *B. rapa,* with 7 non-acylated, 39 mono-acylated, and 19 di-acylated glycosides, respectively.

Remarkable variations of flavonols levels were observed among all genotypes, especially for kaempferol and quercetin glycosides. We found kaempferol 3-O-disinapoylsophorotrioside-7-O-glucoside (compound 235) and kaempferol 3-O-sinapoylsophorotrioside-7-O-glucoside (compound 225) exhibited extremely different levels with 13,123- and 10,432-fold differences between the highest and the lowest values among the tested genotypes, respectively. Similarly, quercetin 3-O-triglucoside-7-O-sinapoylglucoside (compound 258) and quercetin 3-O-triglucoside-7-O-feruloylglucoside (compound 257) displayed 5,577- and 3,549-fold change among all genotypes, respectively. Previous studies have shown that kaempferol derivatives were the most abundant flavonols in Chinese cabbage, pak choi, turnip, and mizuna (Soengas et al., 2018; Dejanovic et al., 2021; Kyriacou et al., 2021; Wiesner-Reinhold et al., 2021), with kaempferol-3,7-di-O-glucoside (compound 176), kaempferol 3-O-caffeoylsophoroside-7-O-glucoside (compound 194), kaempferol 3-O-hydroxyferuloylsophoroside-7-O-glucoside (compound 199), kaempferol 3-O-feruloylsophoroside-7-O-glucoside (compound 201) and kaempferol 3-O-sinapoylsophoroside-7-O-glucoside (compound 205) being the most abundant kaempferol derivatives. These compounds have been reported as antioxidants with high free radical scavenging activity and antimicrobials with effective inhibition of Gram-positive and -negative bacteria (Favela-González et al., 2020; Abellán et al., 2021). In the present study, we found that accessions Chinese cabbage #48, pak choi #10, turnip #88, and mizuna #85 contained the highest levels of these compounds. In addition, Chinese cabbage #48 and pak choi #11 also exhibited high concentrations of isorhamnetin-3,7-di-O-glucoside (compound 168) and isorhamnetin-3-O-glucoside (compound 112). These metabolites have been demonstrated to be active compounds in *Salicornia herbacea* (Lee et al., 2021) and mustard leaf (*Brassica juncea*) (Yokozawa et al., 2002), affecting insulin secretion and blood glucose levels.

The wide variation of flavonols in *B. rapa* determines diverse and important biological functions. Quercetin, kaempferol, and isorhamnetin and their derivatives have diverse bioactivities including antioxidant, antimicrobial, antifungal, and antiviral potentials (Barreca et al., 2021). We found that Chinese cabbage #33 contained the highest amount of isorhamnetin and kaempferol. The highest amount of quercetin was found in another Chinese cabbage accession #35. Previous studies revealed that the caffeoyl moiety due to the O-dihydroxy structure could enhance radical scavenging ability (Braca et al., 2003). In our study, we found 18 caffeoyl kaempferol and quercetin glycosides and one hydroxycaffeoyl kaempferol glycoside. However, glycosylation has been reported to decrease the scavenging activity of flavonoids (De Winter et al., 2015). Considering caffeoyl moiety and glycosylation, kaempferol 3-O-caffeoylsophoroside (compound 182), kaempferol 3-O-caffeoyldiglucoside (compound 183), and quercetin 3-O-caffeoyldiglucoside (compound 244) were expected to be strong antioxidants in *B. rapa* leaves. The highest amounts of compounds 182 and 183 were found in pak choi #11, while Chinese cabbage #63 showed the highest level of compound 244. Therefore, pak choi #11 and Chinese cabbage #63 may be excellent sources of strong antioxidants in *B. rapa*.

#### 3.3.3 Hydroxycinnamic acid derivatives

In *B. rapa*, hydroxycinnamic acid derivatives represent another prominent class of phenolic compounds. The fragmentation of hydroxycinnamic acid glycosides showed the loss of glycosyl and hydroxycinnamoyl moiety to produce hydroxycinnamic acid ions. In the case of hydroxycinnamoyl diglycosides in *Brassica* vegetables, the diglycosyle moiety was mainly characterized as a gentiobiose unit (1→6 glycosidic linkage) (Harbaum et al., 2007; Olsen et al., 2009). For example, compounds 133–136 were detected with a deprotonated molecular ion at *m/z* 739.21. The fragment ion at *m/z* 515.14 was formed by the loss of the sinapoyl (−224.07 amu). The hydroxyferulic acid ion at *m/z* 209.04 was formed by successive loss of gentiobiose moiety (−306.09 amu). Further loss of H2O (−18.01 aum) from hydroxyferulic acid resulted in a fragment ion at *m/z* 191.03. Thus, they were putatively identified as isomers of sinapoyl hydroxyferuloyl gentiobiose. Notably, many isomers of hydroxycinnamic acid derivatives were detected in *B. rapa* leaves (Supplementary Table S1). These isomers could be the result of a different linkage position of the hydroxycinnamoyl group. Some isomers could be distinguished using authentic standards. For example, four caffeoylquinic acid isomers were characterized based on their molecular ion (*m/z* 353.09) and predominant fragment ions (*m/z* 191.06 and 173.04 for quinic acid; *m/z* 179.03, 161.04, and 135.04 for caffeic acid). Finally, three isomers were identified with high confidence as 3-O-caffeoylquinic acid (chlorogenic acid), 5-O-caffeoylquinic acid (neochlorogenic acid), and 4-O-caffeoylquinic acid or 1-O-caffeoylquinic acid (Supplementary Table S1). According to authentic standards and the previously described fragmentation patterns (Harbaum et al., 2007; Lin et al., 2011; Sun et al., 2013), in total 93 hydroxycinnamic acid derivatives were identified in this study, including 4 hydroxycinnamic acids, 4 glycerol and shikimic acid esters, 10 malic acid esters, 14 quinic acid esters, and 61 glycosides. The main derivatives were hydroxycinnamic acid glycosides, including mono-, di-, or triglucose integrated with one, two, or three hydroxycinnamoyl units.

Hydroxycinnamic acid derivatives displayed high variability among the different *B. rapa* studied here. Generally, hydroxycinnamoyl quinic acids accumulated to high levels in all accessions of Chinese cabbage, savoy, pak choi, taicai, and caixin, while they were present at low levels in broccoletto, rapid cycling, yellow sarson, and wild cabbage. Similarly, low levels of hydroxycinnamoyl malic acids were also detected in rapid cycling and yellow sarson. In contrast, hydroxycinnamoyl glycosides were highly abundant in rapid cycling and yellow sarson, as well as in Chinese cabbage and komastuna, but low in caixin, broccoletto, and wild cabbage. According to previous reports (Soengas et al., 2018; Dejanovic et al., 2021), sinapic acid derivatives were the major hydroxycinnamic acid derivatives in *B. rapa*, including 1,2-disinapoyl gentiobiose (compound 147–149) and 1-sinapoyl-2-feruloyl gentiobiose (compound 124–128). These were reported to exhibit antioxidant and anti-inflammatory effects in human plasma and human peripheral blood mononuclear cells (Olszewska et al., 2020). In our study, Chinese cabbage #31 contained the highest amounts of disinapoyl gentiobiose and sinapoyl feruloyl gentiobiose. In another study with pak choi (Heinze et al., 2018) and mizuna (Wiesner-Reinhold et al., 2021), sinapoyl malate was a major hydroxycinnamic acid derivative. Previous studies showed that sinapoyl malate together with other hydroxycinnamoyl malic acids may play an important role in *B. rapa* jasmonate-mediated defense response (Liang et al., 2006). The highest amount of sinapoyl malate was found in mizuna #85. Free hydroxycinnamic acids have been reported to act as powerful antioxidants (Coman and Vodnar, 2020). It was found that hydroxycinnamates work as effective UV-B protectants in *Arabidopsis* (Landry et al., 1995). In this study, four hydroxycinnamic acids were detected in all tested genotypes. The highest amounts of p-coumaric acid and hydroxyferulic acid were found in turnip #92, while pak choi #16 contained the highest levels of sinapic acid and ferulic acid.

#### 3.3.4 Anthocyanins

Anthocyanins are important water-soluble pigments in plants. In the negative ion mode, anthocyanins exhibited a unique doublet of ions [M-2H]^-^ and [M-2H + H2O]^-^ for their molecular ion, which could be used to identify anthocyanins and differentiate them from other polyphenols (Sun et al., 2012). In addition, doubly charged ions were observed for pelargonin and cyanidin glycosides, in some cases as the base peak (Sun et al., 2012). The MS fragmentation of anthocyanins occurred mainly at the glycosidic bonds between the flavylium ring and sugar moieties as well as ester bonds between the sugar moieties and acyl groups (Wu and Prior, 2005). For example, compound 271 was an anthocyanin with the highest level in purple caitai #101. Characteristic doublet ions [M-2H]^-^ and [M-2H + H2O]^-^ were observed at *m/z* 1,239.31 and 1,257.31, respectively. In addition, doubly charged ions at *m/z* 619.14 [M-2H]^2-^ and *m/z* 628.15 [M-2H + H2O]^2-^ were found as the major peaks. The MS fragmentation showed a double-charged ion at *m/z* 597.15 and a single-charged ion at *m/z* 1,195.32 by loss of a carboxyl residue (43.99 amu). In addition, two fragment ions at *m/z* 1,153.30 and *m/z* 991.25 were observed by loss of malonyl residue (86.00 amu) and malonylglucoside moiety (248.05 amu), indicating the presence of a malonylglucoside moiety at the 5-position. Furthermore, successive loss of a feruloyl residue (176.05 amu) and a sinapoyl residue (206.06 amu) gave rise to the fragment ions at *m/z* 609.14, revealing the presence of a feruloyl-sinapoyl residue at the 3-position. Finally, the loss of a diglucose moiety (324.11 amu) from the 3-position produced the cyanidin aglycone ions at *m/z* 285.04 and 284.03. Based on earlier reports (Guo et al., 2015; Song et al., 2020), compound 271 was putatively identified as cyanidin 3-feruloylsinapoylsophoroside-5-malonylglucoside.

All accessions of purple caitai and purple turnip were the only accessions exhibiting purple color due to the presence of anthocyanins. Interestingly, purple caitai only contained cyanidin derivatives, while purple turnip contained exclusively pelargonidin derivatives, indicating different biosynthetic pathways of anthocyanins. In purple caitai, all nine anthocyanins were acylated cyanidin-3-sophoroside-5-glucoside derivatives, as previously shown in *B. rapa* (Guo et al., 2015; Song et al., 2020). However, information on anthocyanin composition in purple turnips was limited so far. In this study, five acylated pelargonidin-3-O-diglucoside-5-O-malonoylglucoside derivatives were putatively identified in purple turnip, which were similar to the anthocyanins reported in red radish (Wu and Prior, 2005; Jing et al., 2014). Anthocyanins have been demonstrated to possess antioxidant activity and preventive activities against cardiovascular disease, metabolism disease, diabetes, and obesity (Ghareaghajlou et al., 2021). The chemical structures of anthocyanins determine their stability, color intensity, and potential biological activity. Previous studies reported that diacylated anthocyanins were characterized by higher antioxidant capacity than monoacylated anthocyanins, while the latter had higher antioxidant capacity than nonacylated forms (Wiczkowski et al., 2013). In addition, acylation with sinapic acid leads to higher antioxidant capacity than with ferulic acid, followed by *p-*coumaric acid (Wiczkowski et al., 2013). In this study, we found that most of the anthocyanins identified in purple caitai and purple turnip contained diacylation with sinapic acid and ferulic acid, modifications that will contribute to good stability and high antioxidant capacity.

#### 3.3.5 Monolignol and oligolignol derivatives

Lignin, an aromatic biopolymer found in plant cell walls, is essential for water transport and mechanical support, and plays an important role in plant defense (Chantreau et al., 2014). Lignin is derived from the combinatorial coupling of monolignol radicals. The MS fragmentation pattern of lignin oligomers has been described previously (Morreel et al., 2010a; Morreel et al., 2010b; Morreel et al., 2014). In this study, we characterized glycosylation and esterification groups, monolignol units, and linkage types. First, for oligolignol glycosides and malate esters, MS fragmentation occurred by loss of glycosyl moiety (324.11 and 162.05 amu) or the malyl moiety (116.01 amu). Second, small neutral losses provide information on the three types of linkages. Third, the first product ions resulting from the cleavage of the linkage yielded the information on the units. For example, compound 298 was detected as a deprotonated ion at *m/z* 581.19. The base peak in MS fragmentation was at *m/z* 419.13 indicating a hexose loss (−162.05 aum). Furthermore, a fragment ion at *m/z* 371.11 was observed that likely resulted from the *β*-aryl ether, a combined loss of water and formaldehyde (−48.02 amu). Fragmentations yield ions at *m/z* 223.06 and 195.06, representing the units derived from sinapic acid and coniferyl alcohol. Furthermore, fragment ions at *m/z* 208.04 and 165.06 indicated a further methyl radical loss from sinapic acid and formaldehyde loss from the G unit, respectively. Therefore, compound 298 was characterized as G(8-O-4)sinapic acid ester hexoside (Supplementary Table S1). In this study, we identified 51 monolignol and oligolignol derivatives in *B. rapa* leaves, including 20 monolignol, 21 lignans and neolignans, and 10 trimeric oligolignols derivatives. To the best of our knowledge, 46 monolignol and oligolignol derivatives are reported here in *B. rapa* leaves for the first time; eight of them were identified with high-confidence levels.

In *B. rapa* leaves, monolignol and oligolignols were mainly composed of guaiacyl (G) and syringyl (S) units that are derived from coniferyl alcohol and sinapyl alcohol, respectively. Various inter-monomeric linkages were observed, including *β*-aryl ether linkage (8-O-4), resinol linkage (8-8), and phenylcoumaran linkage (8-5). In the case of the 8-8 linkage, lignans belonging to different classes were identified, including lariciresinol, pinoresinol, secoisolariciresinol, syringaresinol, and dehydrodiconiferyl alcohol derivatives. Due to the free hydroxyl group from G and S units, most monolignols and oligolignols were glycosylated with one or two hexoses. In addition, monolignol, lignans, and neolignans were conjugated with ferulic acid or sinapic acid, which were further esterified by malate. Recently, a wide range of monolignol and oligolignol derivatives have been found in seed coats of pomegranate (Qin et al., 2020) and arabidopsis leaves (Dima et al., 2015). This indicated that monolignols not only incorporated into lignin polymer biosynthesis/assembly but also participated in other metabolic pathways to form diverse metabolites.

Sinapyl alcohol, the only free monolignol detected in *B. rapa* leaves, exhibited significantly higher amounts in all accessions of oil cabbage, up to 60-fold higher as compared to other genotypes. Monolignol derivatives were detected in higher amounts in all accessions of mizuna and turnip, while the lower amount in oil cabbage, rapid cycling, and yellow sarson. Lignan and neolignan derivatives exhibited a similar accumulation pattern across the accessions, with higher amounts detected in all accessions of mizuna, turnip, and yellow sarson. Trimeric oligolignols derivatives exhibited the highest levels in all accessions of mizuna, turnip, yellow sarson, and broccoletto, while the lowest levels were found in all accessions of oil cabbage. A recent study demonstrated that lignans possess antimicrobial, anti-inflammatory, and antioxidant activities (Hano et al., 2021). Lariciresinol, pinoresinol glucoside (symplocosin), and pinoresinol diglucoside have been proven to possess considerable antioxidant potential in different *in vitro* assays (Gülçin et al., 2006; Soleymani et al., 2020). Here, we found that Chinese cabbage accession #48 contained the highest amount of lariciresinol, while two turnip accessions #87 and #92 contained the highest amount of pinoresinol glucoside and pinoresinol diglucoside, respectively. A previous study showed that lariciresinol glycoside exhibited potent anti-inflammatory activity through the NF-κB signaling pathway (Bajpai et al., 2018). The highest amounts of lariciresinol glycoside were found in Chinese cabbage #48 and rapid cycling #79. Syringaresinol glucoside, an effective regulator of lipogenesis and glucose consumption (Wang et al., 2017), was mainly abundant in mizuna #85 and oil cabbage #78. Finally, some lignans and their glycosides, including secoisolariciresinol, pinoresinol, and lariciresinol, are the precursors for enterolignans with phytoestrogen activity (Hano et al., 2021). Enterolignans are characterized by various biologic activities, including tissue-specific estrogen receptor activation, together with anti-inflammatory and apoptotic effects (Senizza et al., 2020). In this study, the secoisolariciresinol, pinoresinol, and lariciresinol glycoside characterized in *B. rapa* may also be the precursors for the formation of enterolignans (compounds, formed by the action of gut microflora on lignans).

#### 3.3.6 Organic acids and other metabolites

Malic acid, citric acid, and ascorbic acid have been reported as the predominant organic acids in *B. rapa* (Arias-Carmona et al., 2014). Here, we putatively identified ten organic acids in *B. rapa* leaves and they were common in all genotypes. It is well known that malic acid and citric acid contribute to the sensory characteristics due to their sour taste, while ascorbic acid is an important enzyme cofactor, radical scavenger, and donor/acceptor in electron transport (Davey et al., 2000). Malic acid, citric acid, and ascorbic acid showed similar distribution among all genotypes with 2.2-, 6.7-, and 6.1-fold variations between the lowest and the highest levels, respectively. The highest amounts of malic acid, citric acid, and ascorbic acid were detected in Chinese cabbage accession #33, pak choi #16, and a second pak choi accession #10, respectively. The amino acids phenylalanine, tyrosine, and tryptophan were detected in all accessions with 6.1-, 7.8-, and 19.8-fold variations between the lowest and the highest amounts, and the highest amounts were all found in Chinese cabbage #26. Roseoside vomifoliol 9-O-β-D-glucopyranoside (compound 78) was identified with high confidence in *B. rapa* for the first time. This compound characterized before in *Leea aequata* L. showed anticancer activity due to the induction of apoptosis (Rahim et al., 2021). A recent report showed potent anti-inflammatory, antiallergic, and COVID-19 protease inhibitory activities of roseosides (Ebada et al., 2020). In this study, Chinese cabbage #43 had the highest level of vomifoliol 9-O-β-D-glucopyranoside, followed by two additional Chinese cabbage accessions #41 and #52, and turnip #92. Finally, Bn-NCC-1 and Bn-NCC-2, compounds belonging to the tetrapyrroles, were considered potential biomarkers of *Brassica* plants (according to the Dictionary of Natural Products version 30.2 https://dnp.chemnetbase.com) and were both detected in all *B. rapa* genotypes.

### 3.4 Metabolic diversity of the *B. rapa* subspecies and their accessions

**FIGURE 3:**
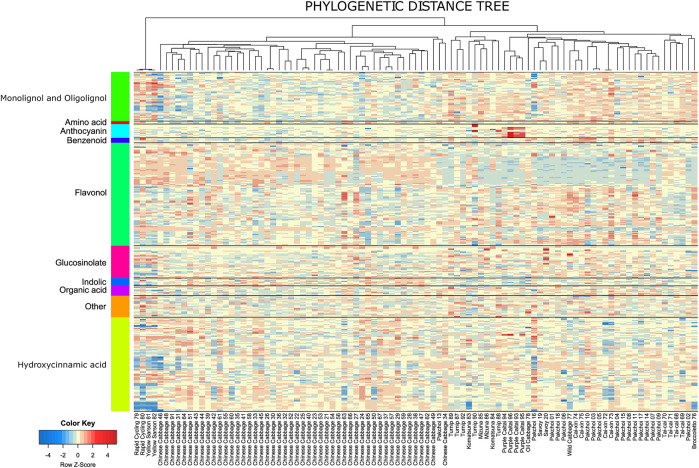
Heatmap of the relative metabolite abundance across the accessions. The heatmap is scaled row-wise (Z-scores from the log values of the metabolite intensity); thus, the colors represent the deviation from the average value obtained for a metabolite. The columns are ordered according to the midpoint-rooted phylogenetic tree.

All except four measured metabolites exhibited significant variation across the genotypes according to ANOVA (FDR-adjusted *p*-value ≤ 0.05; Supplementary Table S2). The only non-significant metabolites included rutin (compound 240), N,O-diacetyl-L-tyrosine (compound 25), and two low-abundant anthocyanins: pg 3-p-coumaroyldiglucoside-5-malonoylglucoside (compound 275) and one of its isomers (compound 277). At the same time, the total contribution of the between-replicate variance was 12% across all measured metabolites. This indicated high specificity of the specialized metabolites composition in measured samples. It appeared that the similarity between metabolic profiles across the *B. rapa* genotypes of the same subspecies is much higher than the similarity between the subspecies (Figure 3). This concerns practically all measured metabolite classes, but most remarkably anthocyanins, flavonols, and hydroxycinnamic acid derivatives. For example, anthocyanins are found in high levels in all Purple caitai accessions. Several flavonols are specifically accumulated in all Chinese cabbage accessions but were found at low levels in pak choi, caixin, savoy, turnip, and mizuna accessions. Yellow sarson and rapid cycling accessions on the other hand accumulated a group of lignans and several specific hydroxycinnamic acid derivatives.

Knowing the phylogenetic relationship and quantitative phenotype traits, across a population, it is possible to quantify how much the value of a certain trait is related to the phylogeny. In the case of the analyzed *B. rapa* subspecies and their accessions, the evolutionary process is represented by agronomical trait selection and the traits of interest are levels of measured biochemical compounds. We explored this phenomenon in a systematic way enumerating the trait-phylogeny relationship.

Phylogenetic signal, a quantitative measure of the trait-phylogeny relationship (Hillis and Huelsenbeck, 1992), has been estimated for the metabolic profiles using the phylogenetic neighbor-joining tree calculated from all annotated polymorphisms. Specifically, we applied a well-established method of Blomberg et al. (2003), using a Brownian motion model to simulate the evolution of the traits along the branches of the phylogenetic tree. The distribution of the metabolic traits, for example, relative accumulation of each metabolite, compared with the simulated model provides an informative statistical output in terms of comparable K statistic values. The method is adequate for rough phylogenetic relatedness estimation using the SNP-based neighbor-joining tree, as it was shown to be robust against errors likely emerging in branch length estimation (Münkemüller et al., 2012). For a test of significance estimation, both the theoretical and empirical *p*-values have been computed. Due to the sensitivity of the K statistics to the differences in the trait values distribution, here we used only empirical *p*-values derived from the 999-fold permutation test (results attached in Supplementary Table S3).

**FIGURE 4:**
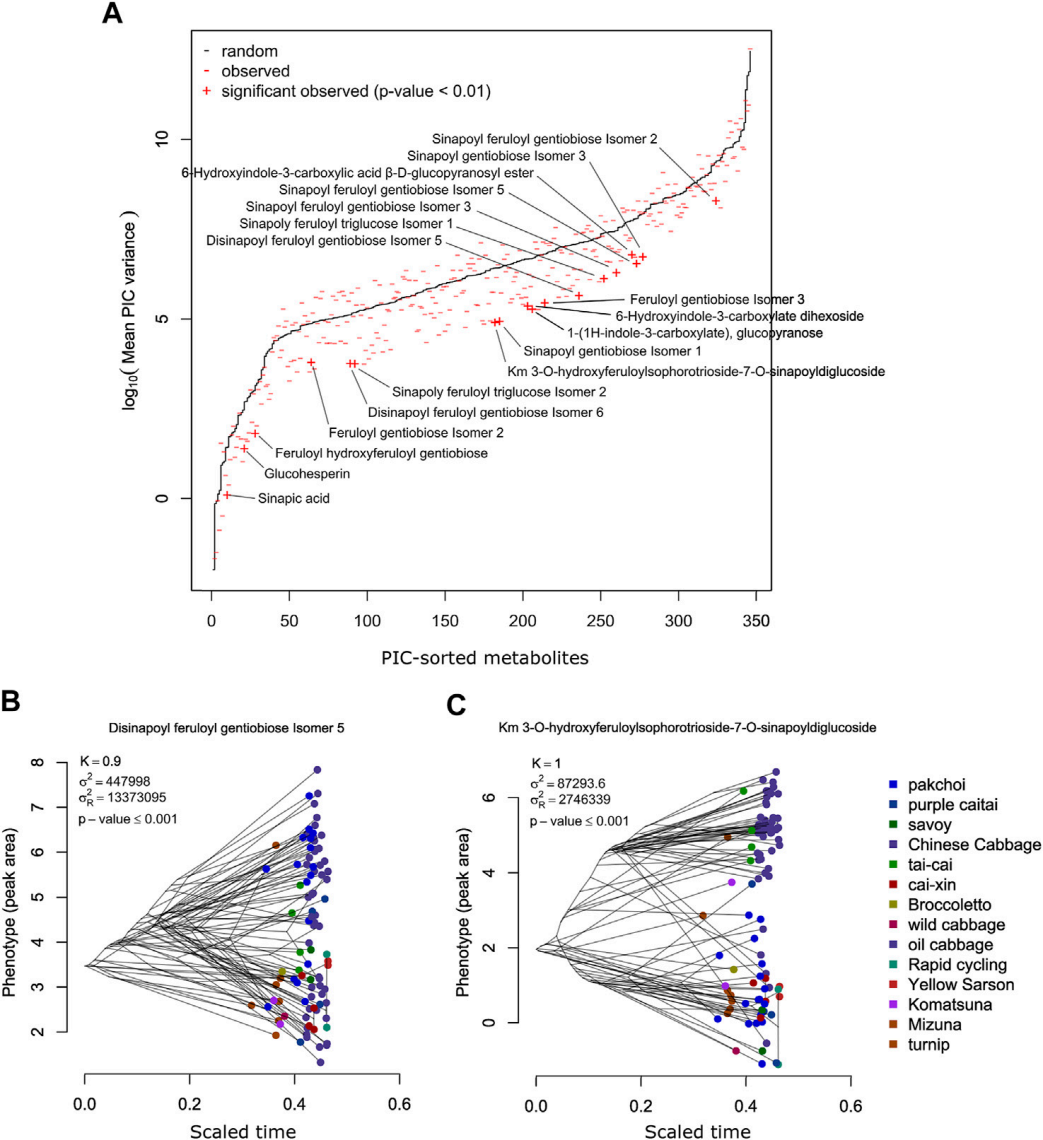
**(A)** Comparison of the PIC values expected from the random Brownian motion model (black) and PIC observed for the measured metabolites. Metabolites exhibiting significantly lower PIC (*p*-value ≤ 0.01) are labeled. **(B)** Phenogram of the disinapoyl feruloyl gentiobiose isomer 5—an example of a metabolite exhibiting high and significant phylogenetic signal. **(C)** Phenogram of the km 3-O-hydroxyferuloylsophorotrioside-7-O-sinapoyldiglucoside—another example of a metabolite exhibiting high and significant phylogenetic signal.

A comparison between observed and randomized data is shown in Figure 4A. PIC variance value (standardized phylogenetic independent contrast scaled by the branch length) is a measure reflecting how the independence of the trait values decreases with the decreasing phylogenetic distance. The PIC for randomized data without scaling is affected by the total trait variance and thus individual tests were performed for each metabolite. In Figure 4A, results for individual metabolites were sorted according to the average PIC obtained for 999 random permutations. In general, observed PIC values are shifted towards lower values with respect to the mean value obtained in 1,000 random permutations (red marks are mostly below the black line); however, only some of them deviate significantly. In Figure 4A, 18 metabolites with an empirical *p*-value ≤ 0.01 have been highlighted.

**FIGURE 5:**
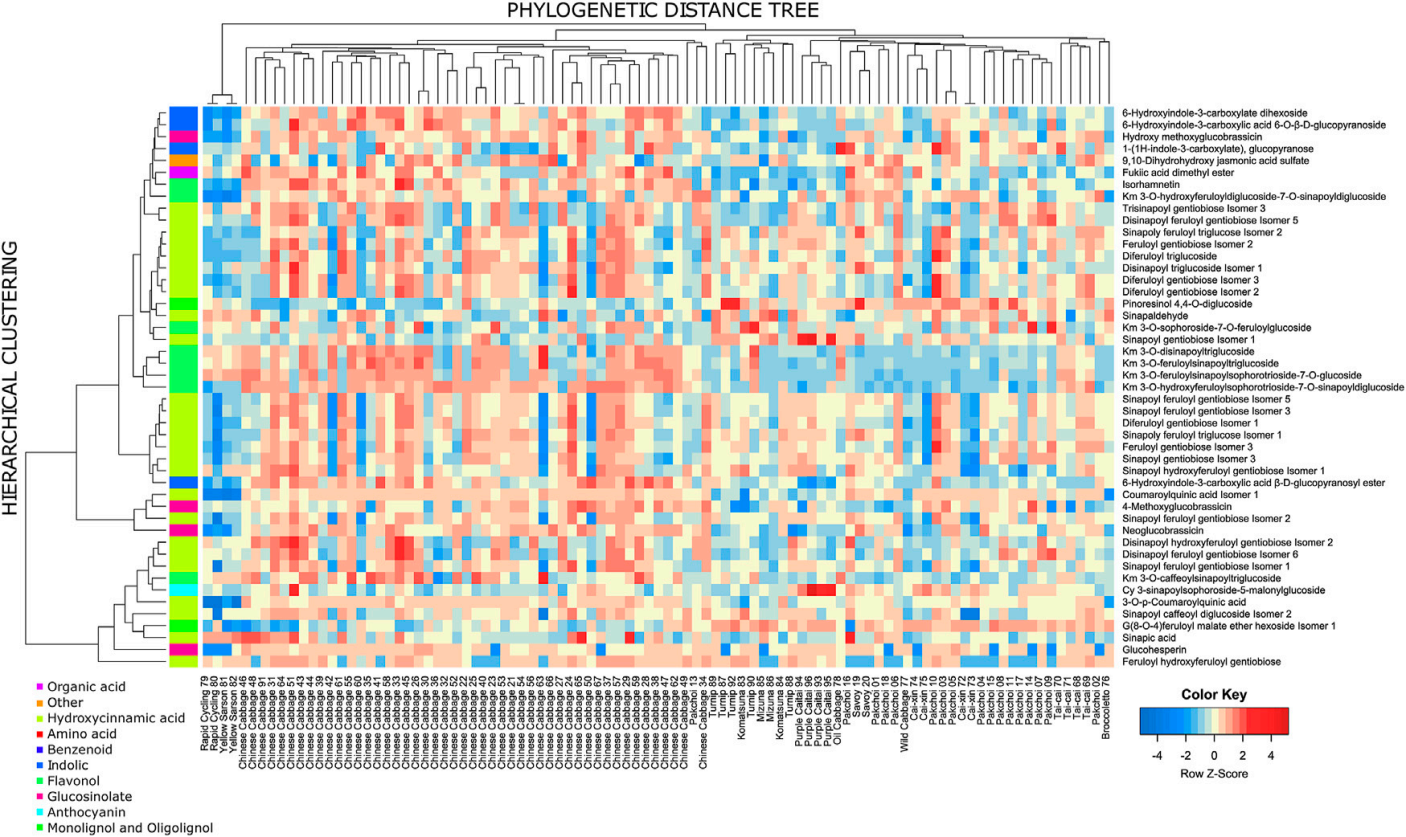
Heatmap of compounds exhibiting significant phylogenetic signal (*p*-value ≤ 0.05). The side color sidebar represents the classification of measured compounds to 10 biochemical classes. The row-wise clustering tree is based on the Euclidean distance between Z-transformed metabolite levels, and the average linkage method for cluster agglomeration. The column tree is a midpoint-rooted phylogenetic tree.

Among the 346 measured metabolites, 18 metabolites exhibited a significant phylogenetic signal with a *p*-value ≤ 0.01 and 47 with a *p*-value ≤ 0.05. To visualize the connection between metabolite level and phylogeny, we show phylograms of two highly significant metabolites: disinapoyl feruloyl gentiobiose isomer 5 (compound 160) and kaempferol 3-O-hydroxyferuloylsophorotrioside-7-O-sinapoyldiglucoside (compound 239) (Figures 4B, C). In a phylogram, branches of a phylogenetic tree are organized according to their phenotype value (*y*-axis) and standardized time of the modeled evolutionary process (*x*-axis). We observed that the phylogenetic branches of Chinese cabbage are shifted towards higher levels of both metabolites, whereas, for example, pak choi and yellow sarson are much lower. There are also differences between both metabolites, whereas for compound 160, pak choi accessions exhibited a wide range of metabolite accumulation, overlapping with the Chinese cabbage; in the case of compound 239, pak choi accumulated much lower levels than the Chinese cabbage. The 47 compounds that have been selected as exhibiting significant phylogenetic signals with an empirical *p*-value ≤ 0.05 are enriched in hydroxycinnamic acid derivatives and indolics (Figure 5; Fisher’s exact test *p*-values ≤ 0.01). Most of the significant metabolites are described by the differential accumulation in four major phylogenetic branches: 1) the Chinese cabbage, 2) the yellow sarson and rapid cycling, 3) the mizuna-komatsuna-turnip-caitai branch, and 4) the rest of the genotypes. This separation highlights the major metabolic effects of the selection pressure, leading to the development of modern *B. rapa* subspecies and their individual accessions. It is also an indication that the stepwise changes in specialized metabolism during *B. rapa* selective breeding processes are observable and can be reconstructed from metabolomics data. Finally, it is important to note that while the estimated phylogeny stays in concordance with the population structure (Supplementary Figure S6), it is only a rough approximation of the evolutionary process leading to the emergence of the analyzed genotypes. At this point, analysis of the evolution of particular metabolic traits and identification of evolutionary events and loci associated with the accumulation of specific metabolites requires the inclusion of more genotypes and the association mapping with higher statistical power.

## 4 Conclusion

This study presents comprehensive metabolite profiling of *B. rapa* leaves from 102 different genotypes. By this approach, a total of 346 metabolites were identified. Among them, 36 metabolites were identified in high confidence, and 184 metabolites were reported in *B. rapa* leaves for the first time. HCA and phylogenetic analysis were applied to reveal metabolite diversity and accumulation patterns as well as to identify species-specific metabolites. This work expanded the current information on *B. rapa* metabolites. It provides valuable information for developing new *B. rapa* accessions with high levels of selected metabolites possessing health-promoting activity or desired physiological function. The analysis also exemplified how selective pressure in agriculture might utilize the native biosynthetic capacity of the species to achieve highly divergent metabolic phenotypes.

## Data Availability

Original datasets with respective metadata and methods are available in a publicly accessible e!DAL repository (Arend et al. 2016): https://doi.org/10.5447/ipk/2022/27.
