# Supplementary material for: Metabolic diversity in a collection of wild and cultivated Brassica rapa subspecies
Source: Front Mol Biosci. 2022 Nov 16;9:953189. doi: 10.3389/fmolb.2022.953189 (PMC9709217; doi:10.3389/fmolb.2022.953189)
Supplement: Supplementary file 1 [file DataSheet1.zip › Supplementary materials.PDF]

<u>Supplementary material</u>

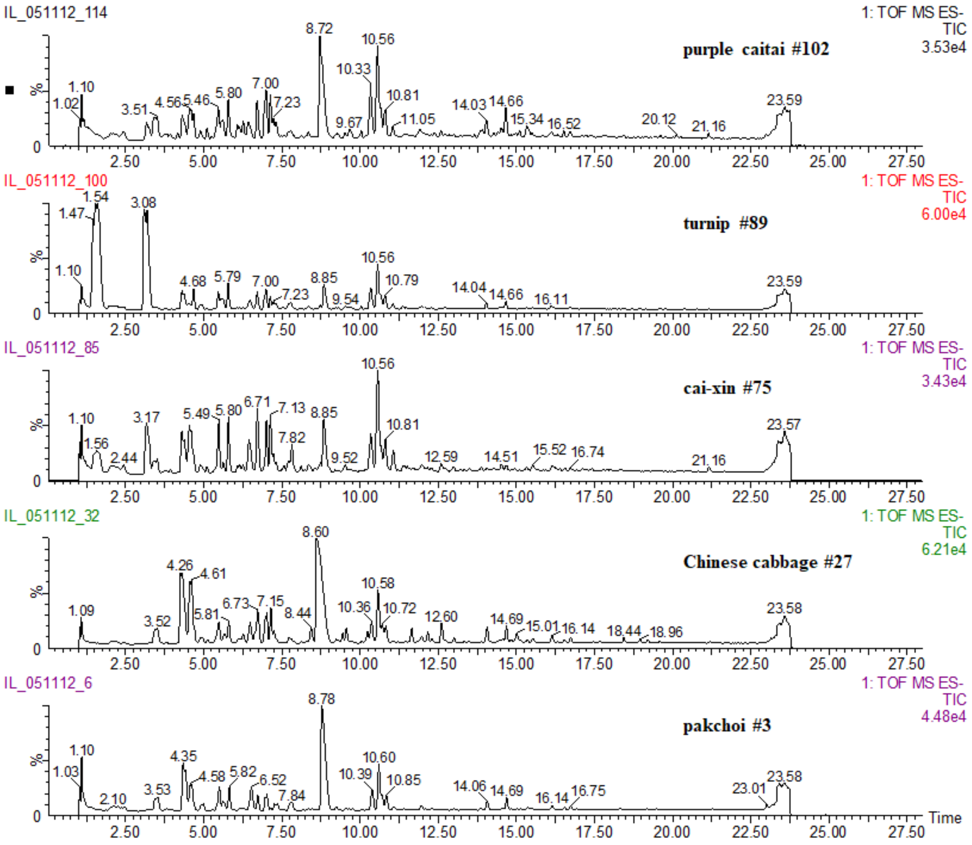

Fig. S1. Total ion chromatograms of purple caitai #102, turnip #89, cai-xin #75, Chinese cabbage #27 and pack choi #3.

Metabolite isomers identification and differentiation

In this study, metabolites isomers were found in *B. rapa* leaves and they were identified and differentiated based on retention time and/or MS fragment ions. We have chosen two isomeric glucosinolates (compound 110 and 111), two isomeric flavonols (compound 181 and 245) and four caffeoylquinic acid isomers (compound 59-62), as examples to explain the metabolite isomers identification and discrimination in detail.

Example 1: two isomeric glucosinolates (compounds 110 and 111)

Extracted ion chromatograms of compounds 110 and 111 with retention time 6.52 min and 8.74 min are shown in Fig. S2a, showing complete chromatographic separation of the two isomers. They exhibited the same deprotonated molecular ion at *m/z* 477.06 (Fig. S2b-c) and formula as $C_{17}H_{22}N_2O_{10}S_2$. Both fragment spectra (Fig. S2d-e) show glucosinolate-characteristic fragment ions at *m/z* 96.96 (sulfate anion) and *m/z* 259.01 (sulfated glucose anion), indicating that they were two isomeric glucosinolates. By surveying the literature and public databases, these two isomers were assigned as 4-methoxyglucobrassicin and neoglucobrassicin, with the structure difference only in substituent positions of the methoxy group. Due to loss of methoxy group via the cleavage of N-O chemical bond between the methoxy group and indole ring, neoglucobrassicin gave rise to a specific fragment ion at *m/z* 446.04, which was observed in compound 111 fragmentation (Fig. S2e). Therefore, compound 111 was identified as neoglucobrassicin and differentiational from compound 110 assigned as 4-methoxyglucobrassicin.

a

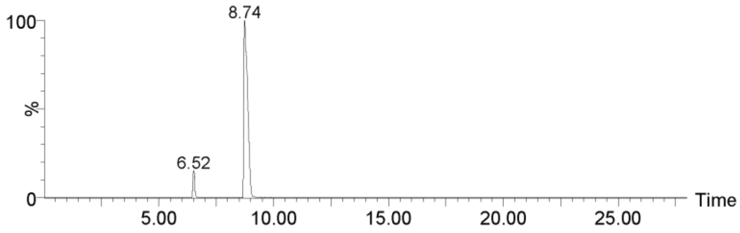

b

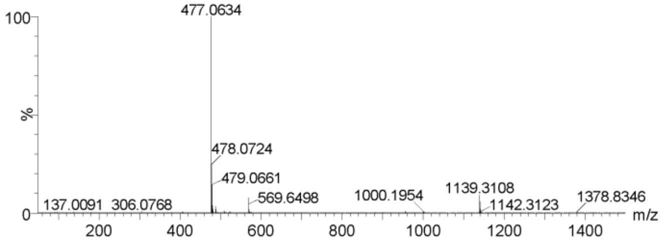

c

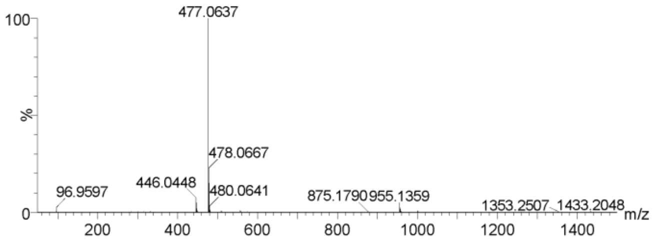

d

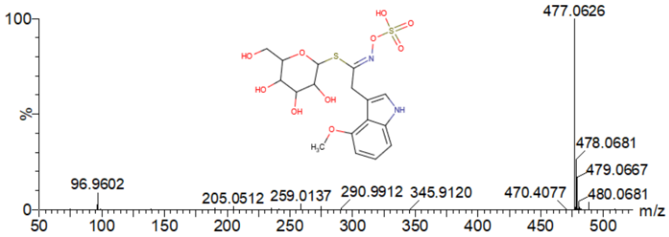

e

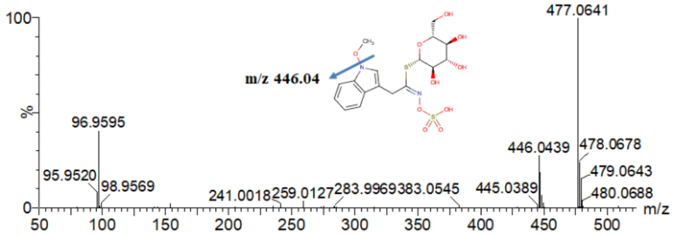

Fig. S2. Chromatograms and MS spectra of compounds 110 and 111. **a** Extracted ion chromatograms of compounds 110 and 111 at *m/z* 477.06 from Chinese Cabbage # 64; **b-c**, MS spectra at low collision energy for compounds 110 and 111; **d-e**, MS spectra at high collision energy for compounds 110 and 111.

Example 2: two isomeric flavonols (compound 181 and 245)

Compounds 181 and 245 exhibited the same deprotonated molecular ion at *m/z* 801.19 (Fig. S3b-c) and formula as $C_{37}H_{38}O_{20}$. They were eluted with different retention time at 9.45 min and 10.22 min, respectively. Extracted ion chromatograms are shown in Fig. S3a, showing complete chromatographic separation of these two isomers. From fragment spectrum of compound 181 (Fig. S3d), a main fragment ion at *m/z* 609.15 was observed due to loss of a hydroxyferuloyl moiety (-192.04 amu). Further loss of diglucosyl moiety (-324.11 amu) at the 3-position gave rise to the kaempferol aglycone ions at *m/z* 285.04. Moreover, the fragment ions at *m/z* 429.08 (-180.06 amu) and *m/z* 489.10 (-120.04 amu) were observed to confirm the diglucosyl moiety at the 3-position as sophorosyl moiety. Thus, compound 181 was putatively identified as kaempferol 3-O-hydroxyferuloylsophoroside. For compound 245, a main fragment ion at *m/z* 625.14 was observed (Fig. S3e) due to loss of a feruloyl moiety (-176.05 amu) at the 3-O position. After loss of a diglucosyl moiety at the 3-position (-324.11 amu), quercetin aglycone ions at *m/z* 300.03 were detected. Moreover, fragment ions at *m/z* 505.11 (-120.04 amu) and *m/z* 445.07 (-180.06 amu) to confirm the sophorosyl moiety. Thus, compound 181 was putatively identified as quercetin 3-O-feruloylsophoroside.

a

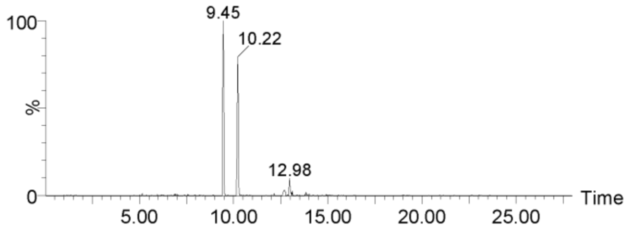

b

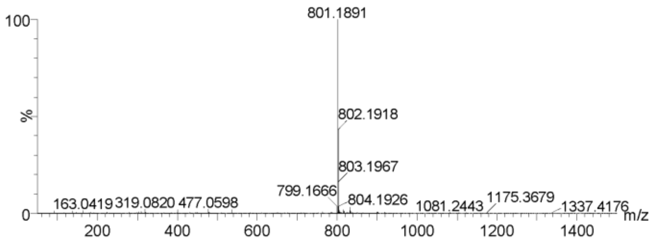

c

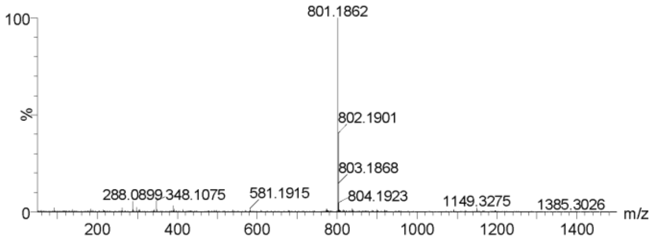

d

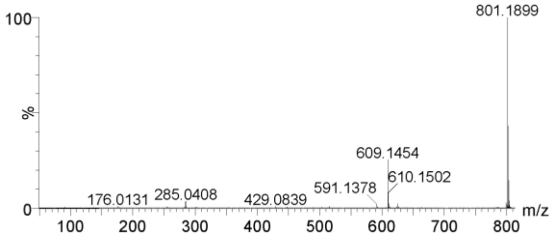

e

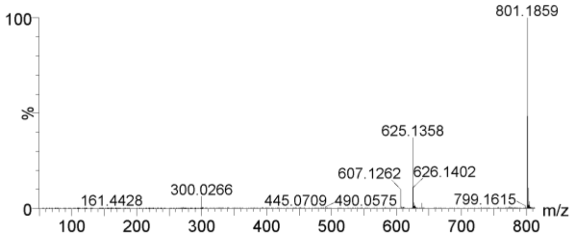

Fig. S3. Chromatograms and MS spectra of compounds 181 and 245. **a** Extracted ion chromatograms of compounds 181 and 245 at *m/z* 801.19 from Chinese Cabbage # 27; **b-c**, MS spectra at low collision energy for compounds 181 and 245; **d-e**, MS spectra at high collision energy for compounds 181 and 245.

Example 3: four caffeoylquinic acid isomers (compounds 59-62)

Notably, many isomers of hydroxycinnamic acid derivatives were detected in B. rapa leaves (Supplementary Table 1). These isomers could be the result of a different linkage position of the hydroxycinnamoyl group. Some isomers could be distinguished using authentic standards. For example, compounds 59-62 were detected with the same deprotonated molecular ion at *m/z* 353.09 (Fig. S4b) and assigned the same formula as $C_{16}H_{18}O_9$. They were completely chromatographic separated with different retention time at 4.97, 3.53, 5.47 and 6.65 min, respectively (Fig. S4a). Characteristic fragment ions (*m/z* 191.06 and 173.04 for quinic acid; *m/z* 179.03, 161.04 and 135.04 for caffeic acid) were observed in their

fragment spectra (Fig. S4c-f), indicating they are caffeoylquinic acid isomers. Three of them were identified with well-defined linkage position of the hydroxycinnamoyl group, as chlorogenic acid (compound 59), neochlorogenic acid (compound 60) and 4-O-caffeoylquinic acid or 1-O-caffeoylquinic acid (compound 61), based on the comparison of retention time and fragment ions with available authentic standards. Because 4-O-caffeoylquinic acid and 1-O-caffeoylquinic acid, two standards were coeluted in this LC method and they had the same fragments, these two isomers were not differed in our method. The last isomer was assigned as caffeoylquinic acid (compound 62) without clear linkage position of the hydroxycinnamoyl group due to unavoidable standards.

a

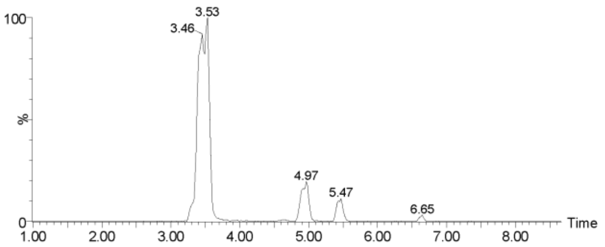

b

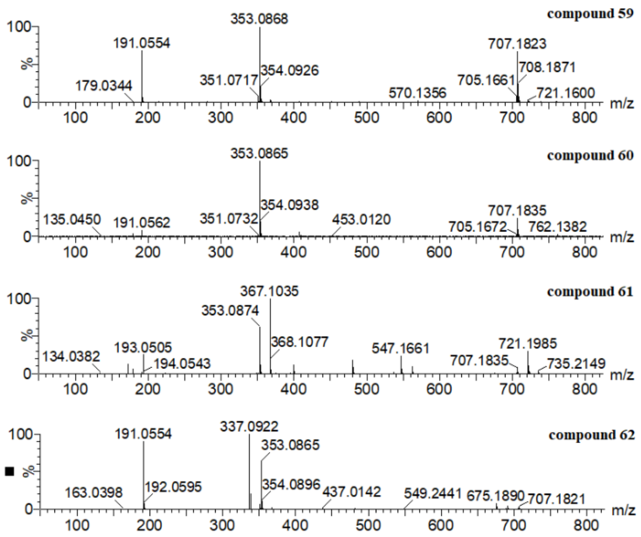

c

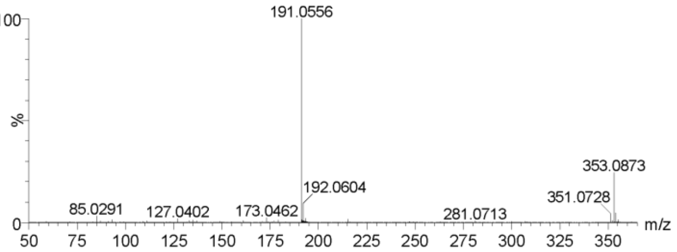

d

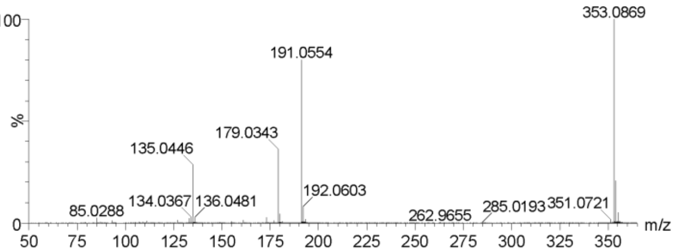

e

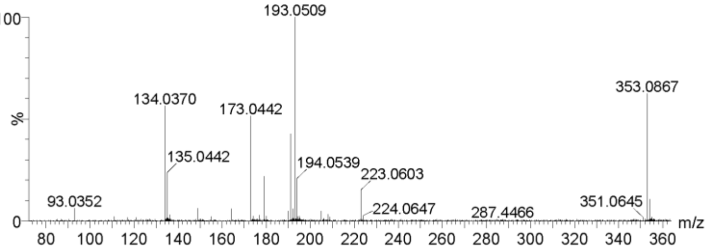

f

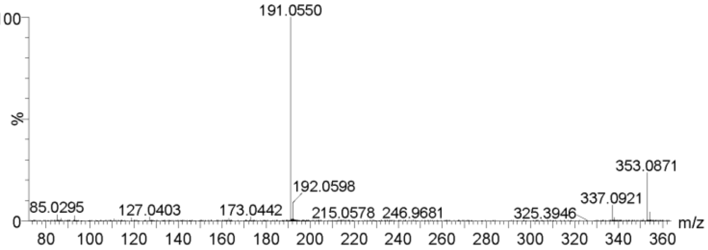

Fig. S4. Chromatograms and MS spectra of compounds 59-62. **a** Extracted ion chromatograms of compounds 59-62 at *m/z* 353.09 from pak choi # 14; **b**, Mass spectra at low collision energy for compounds 59-62; **c-f**, Mass spectra at high collision energy for compounds 59-62.

Identification of compound 263

Compound 263 eluted at 10.97 min (Fig. S5a) and detected $[M-H]^-$ ion at m/z 1317.34 and $[M-2H]^{2-}$ at *m/z* 658.16 as base peak (Fig. S5b), assigned chemical formula as $C_{59}H_{66}O_{34}$. The fragment spectrum (Fig. S5c) showed two fragment ions at *m/z* 1155.28 with single charge and *m/z* 577.13 with double charge due to loss of a glucosyl moiety (-162.05 amu) at the 7-O position. Further loss of caffeoyl moiety (-162.03 amu) and sinapoyl moiety (-206.06 amu) at the 3-position gave rise to the fragment ions at *m/z* 993.25 and *m/z* 949.23, respectively. Besides, simultaneous loss of caffeoyl moiety (-162.03 amu) and sinapoyl moiety (-206.06 amu) at the 3-position gave the fragment ion at *m/z* 787.19. After loss of a triglucosyl moiety at the 3-position (-486.16 amu), quercetin aglycone ions (*m/z* 300.03 and *m/z* 301.03) were detected. Because unavailable information of interglycosidic linkage for triglucosyl moiety at the 3-position, compound 263 was putatively identified as quercetin 3-O-caffeoylsinapoyltriglucoside-7-O-glucoside.

a

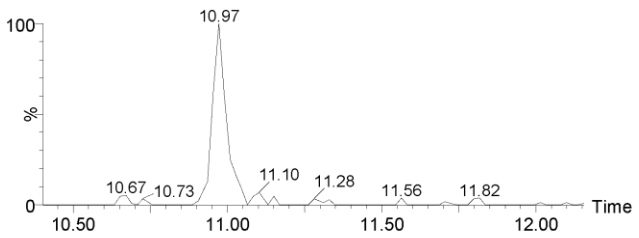

b

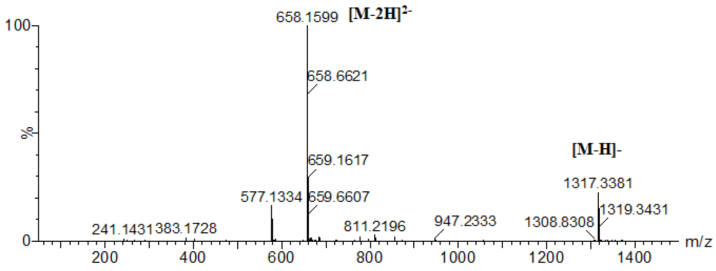

c

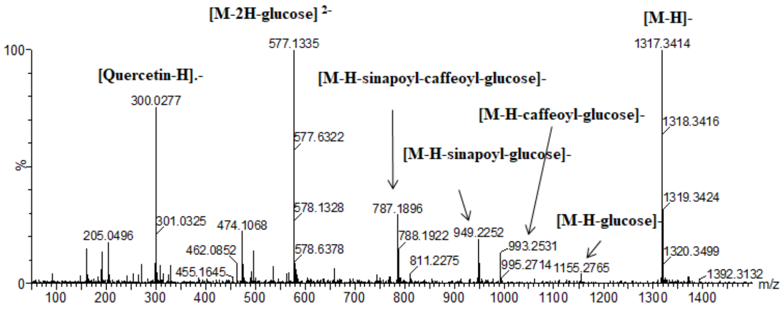

Fig. S5. Chromatogram and MS spectra of compound 263. **a** Extracted ion chromatogram of compound 263 at *m/z* 1155.28 from Yellow Sarson # 81; **b**, Mass spectrum at low collision energy for compound 263; **c**, Mass spectrum at high collision energy for compound 263.
