# Supplementary figures and images for: Metabolic diversity in a collection of wild and cultivated Brassica rapa subspecies
Source: Front Mol Biosci. 2022 Nov 16;9:953189. doi: 10.3389/fmolb.2022.953189 (PMC9709217; doi:10.3389/fmolb.2022.953189)

# PCoA ordination

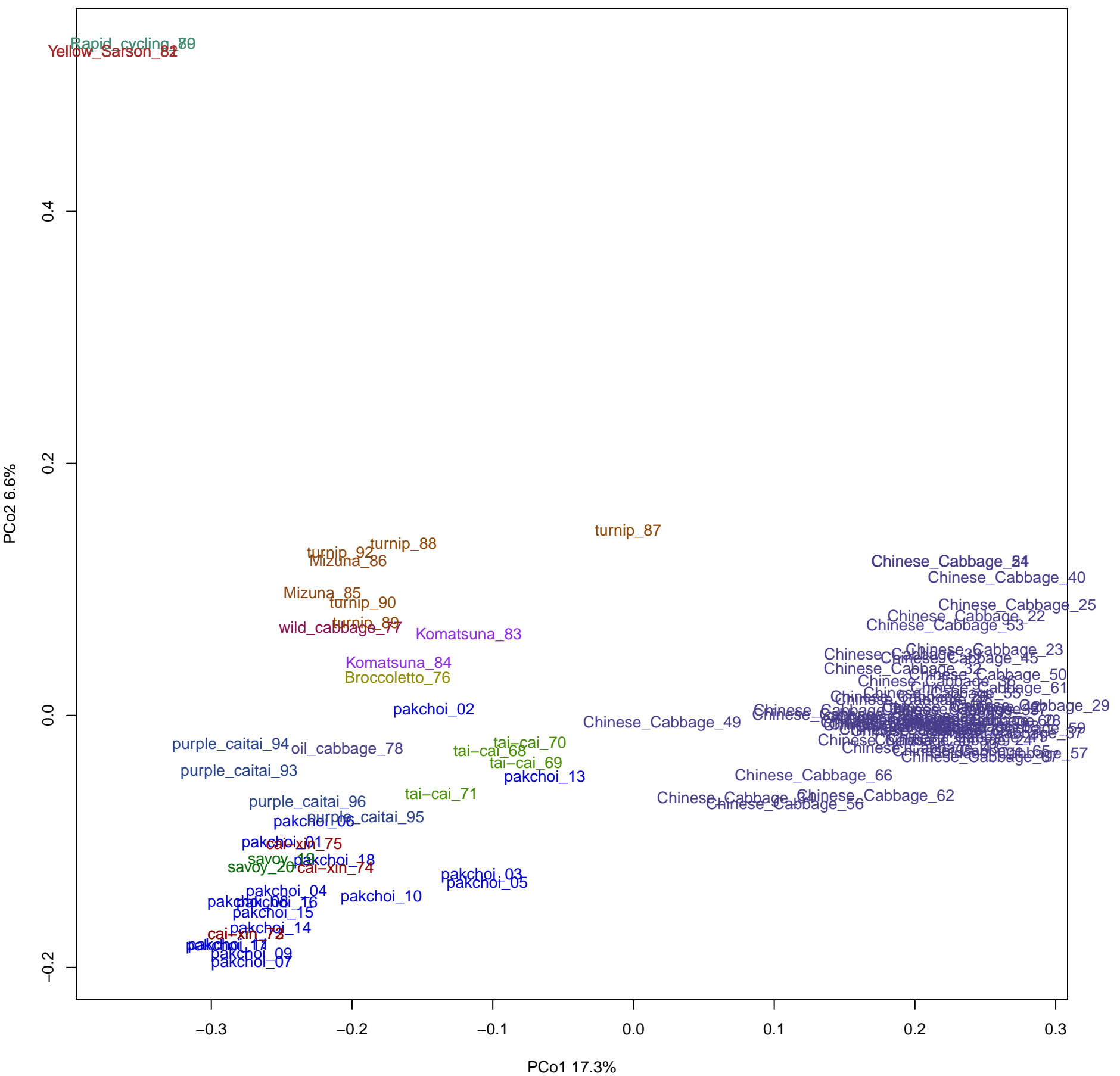

## PCoA ordination

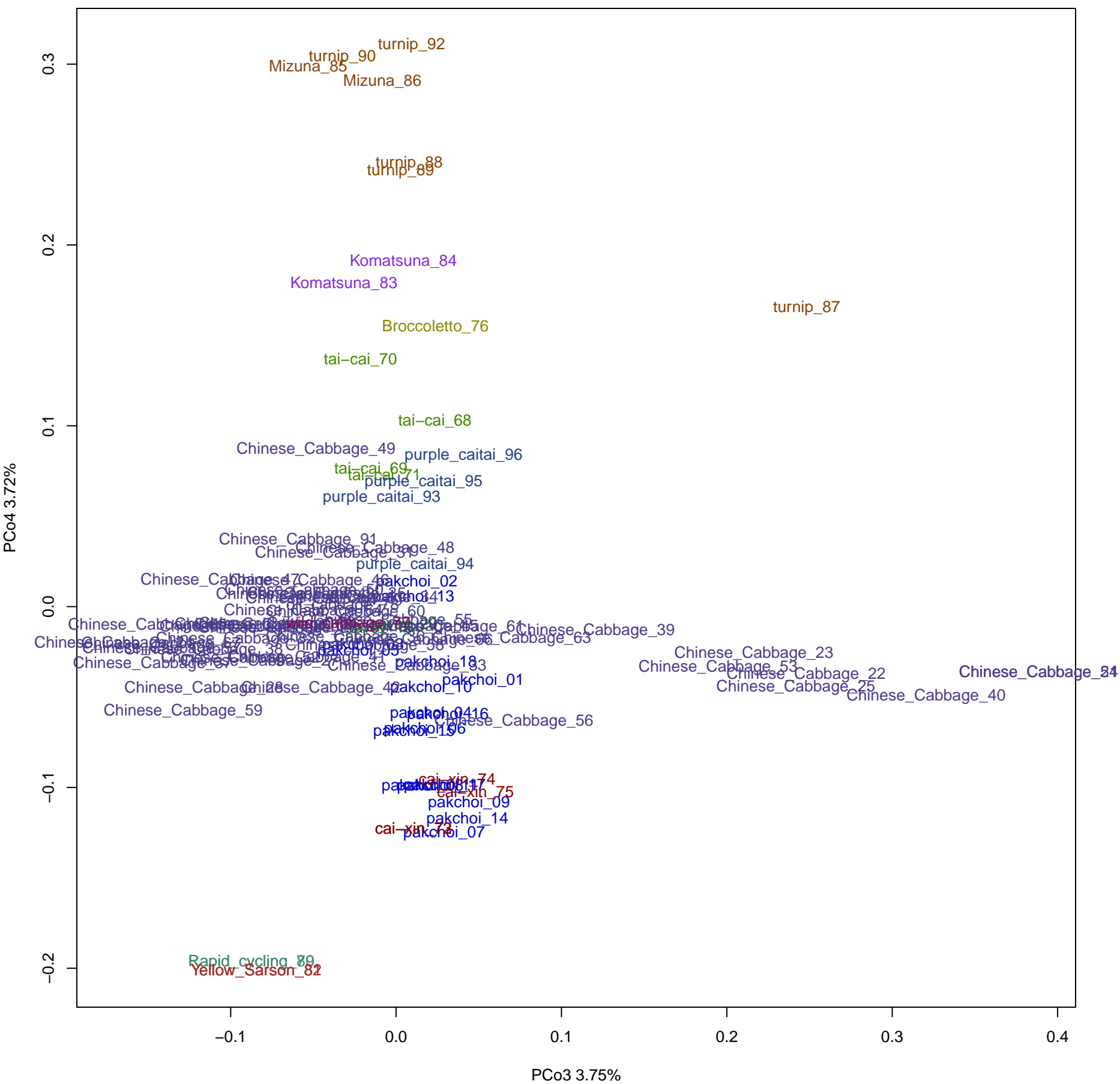

# PCoA ordination

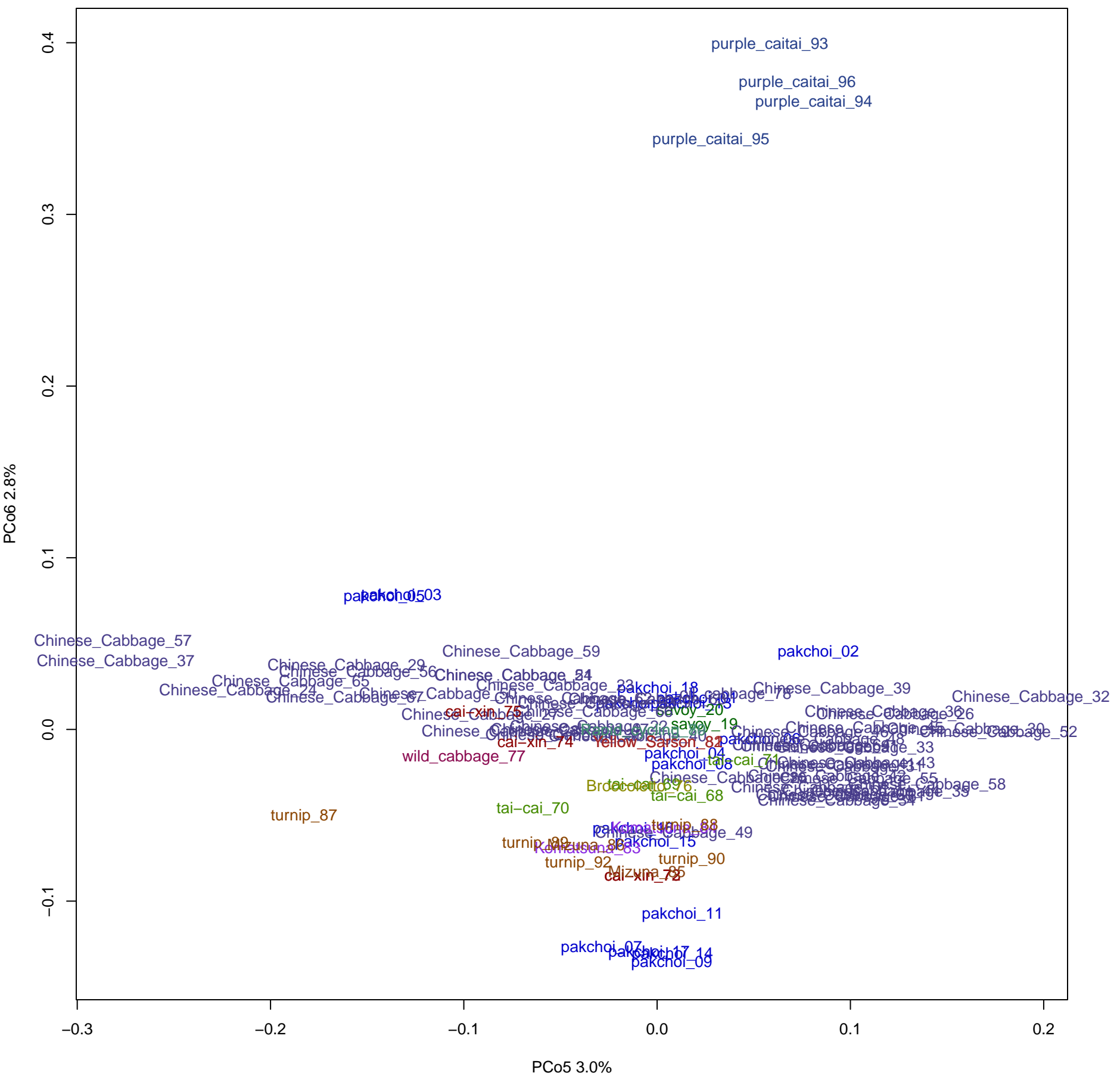

Supplement: Supplementary file 1 [file DataSheet1.zip › Supplementary Figure S6.PDF]
